# Epidemiology of pulmonary disease due to nontuberculous mycobacteria in Southern China, 2013–2016

**DOI:** 10.1186/s12890-018-0728-z

**Published:** 2018-11-09

**Authors:** Yaoju Tan, Biyi Su, Wei Shu, Xingshan Cai, Shaojia Kuang, Haobin Kuang, Jianxiong Liu, Yu Pang

**Affiliations:** 1 Department of Clinical Laboratory, Guangzhou Chest Hospital, State Key Laboratory of Respiratory Disease, No. 62, Hengzhigang Road, Yuexiu District, Guangzhou, Guangdong Province 510095 People’s Republic of China; 2 National Clinical Laboratory on Tuberculosis, Beijing Key laboratory on Drug-resistant Tuberculosis Research,, Beijing Chest Hospital, Beijing Tuberculosis and Thoracic Tumor Institute, No. 9, Beiguan Street, Tongzhou District, Beijing, 101149 People’s Republic of China

**Keywords:** Nontuberculous mycobacteria, Epidemiology, Slowly growing mycobacteria, Rapidly growing mycobacteria, Comorbidity

## Abstract

**Background:**

Pulmonary nontuberculous mycobacteria (NTM) disease is of increasing public health concern in China. Information is limited regarding risk factors associated with this disease in China. The objective of this study was to describe the epidemiology of pulmonary disease due to NTM in Southern China.

**Methods:**

We retrospectively reviewed the medical records of pulmonary NTM patients registered in the Guangzhou Chest Hospital with positive mycobacterial cultures during 2013–2016. We described sex, age, residence, treatment history, laboratory examination results and comorbidities of pulmonary NTM patients.

**Results:**

Among the 607 NTM cases, the most prevalent species were *Mycobacterium avium* complex (44.5%), *Mycobacterium abscessus* complex (40.5%), *Mycobacterium kansasii* (10.0%) and *Mycobacterium fortuitum* (2.8%). The male:female ratio was significantly lower among patients infected with rapidly growing mycobacteria (RGM) than among those with slowly growing mycobacteria (SGM). The risk of developing SGM disease significantly increased with advancing age. In addition, pulmonary RGM diseases were more common in migrant population than resident population. Notably, patients with pulmonary RGM diseases were significantly more likely to have bronchiectasis underlying noted than those with SGM diseases. No significant difference was observed in in vitro drug susceptibility among NTM species.

**Conclusion:**

Our data illustrate that the *M. avium* complex is the most predominant causative agent of pulmonary NTM disease in Southern China. Female, migrant population, the presence of bronchiectasis are independent risk factors for pulmonary diseases due to RGM. In addition, the prevalence of SGM increases significantly with advancing age.

## Background

Nontuberculous mycobacteria (NTM) are a heterogeneous group of species other than the *Mycobacterium tuberculosis* complex and *Mycobacterium leprae* [1]. As the etiologic agents, NTM have been found in a variety of environmental sources, such as soil, water and aerosols [2]. Despite being less pathogenic than *M. tuberculosis*, these environmental bacteria are associated with a wide array of clinical diseases, especially in HIV-infected patients or those with immunodeficiencies [3]. Notably, NTM disease incidence has increased significantly during the past decade [3], while this emerging disease is given a lower public health priority as compared with tuberculosis due to lack of definitive evidence of person-to-person transmission of NTM [4]. The most available data on NTM infections come from sentinel laboratory–based surveillance studies [5, 6], which makes it difficult to distinguish between colonizers and causative pathogens among these positive mycobacteria cultures [7]; consequently, the exact distribution of mycobacteria species among patients is not well known, especially in high-TB-burden settings.

Although China has achieved impressive reductions in TB prevalence and mortality over the past 20 years [8], NTM infections have become a serious issue, accounting for about one quarter of mycobacterial patient isolates according to the national population-based data [9], triggering public health concerns. More importantly, NTM prevalence varies greatly across China, and Southern China has a significantly higher proportion of NTM infection [1]. In addition, national epidemiological data have revealed that NTM species distribution differs significantly by region, reflecting the diversity of species distribution in the local environment [3]. Given that NTM species differ significantly in pathogenicity and drug susceptibility profiles, understanding this regional diversity is a major priority for optimizing appropriate treatment regimen. Unfortunately, previous reports regarding this issue are lacking, and few laboratory data were mainly on the basis of NTM isolates from microbiology laboratory, which made it difficult to differentiate NTM diseases from host respiratory colonization [1, 10]. Furthermore, information is limited regarding risk factors associated with this disease, thereby hampering attempts to implement effective infection control programmes. The objective of this study was to describe the prevalence of NTM species among pulmonary NTM patients in regional tuberculosis clinical centre in Southern China between 2013 and 2016. We also aimed to identify demographic and clinical factors associated with pulmonary NTM diseases between slowly growing mycobacteria (SGM) and rapidly growing mycobacteria (RGM).

## Methods

### Study design and population

This study was conducted at the Guangzhou Chest Hospital, an 800-bed regional tuberculosis clinical centre in Southern China. We retrospectively reviewed the medical records of pulmonary NTM patients registered in the hospital with positive mycobacterial cultures during 2013–2016. Factors that were assessed in this study included demographic and clinical characteristics, such as sex, age, residence, treatment history, laboratory examination results and comorbidities. The definition of NTM lung disease met the criteria established by the American Thoracic Society (ATS) in 2007 [11], including clinical symptoms and abnormal chest radiograph suggestive of pulmonary TB or NTM diseases; isolation of the same NTM species from more than two sputum specimens collected at different time points; and exclusion of other differential diagnoses. In addition, the residents were defined as individuals with the local household registration of Guangzhou, while the migrants were defined as individuals without local household registration of Guangzhou.

### Laboratory examination

Media supplied with paranitrobenzoic acid was used for differential identification of *Mycobacterium tuberculosis* (MTB) complex and NTM. The NTM strains identified by conventional biochemical method were subcultured on the Löwenstein-Jensen (L-J) medium [1]. Colonies were scraped from the surface of L-J medium, and transferred to 500 μL Tris-EDTA (TE) buffer. The was heated at 95 °C for 30 min in a water bath, and the supernatant was used as DNA template for PCR amplification. The commercial Biochip test was performed for species identification of mycobacterium according to the manufacturer’s instructions [12]. In addition, the isolates identified as *Mycobacterium chelonae*-*Mycobacterium abscessus* group by Biochip were further divided into subspecies with the sequencing of multiple genes, including 16S rRNA, *hsp65*, *rpoB*, and 16S–23S rRNA internal transcribed spacer (ITS) sequence as previously reported [9]. The PCR products were sent to Ruibo Company (Beijing, China) for DNA sequencing service. Nucleotide sequences were aligned with the homologous sequences of the reference mycobacteria strains by using multiple sequence alignments via the BLAST web pages (http://www.ncbi.nlm.nih.gov/BLAST).

### Drug susceptibility testing

The in vitro drug susceptibility of *M. abscessus* complex was determined with a broth microdilution method based on the guidelines from the Clinical and Laboratory Standards Institute (CLSI) [13]. Eight antimicrobial agents were enrolled in this study, including amikacin, clarithromycin, linezolid, tobramycin, cefoxitin, ciprofloxacin, doxycycline and imipenem. The breakpoint values to distinguish susceptibility and resistance for drugs were followed as recommendation from CLSI [13]. For *M. avium* and *M. intracellulare* isolates, three agents were selected for MIC assessment, including clarithromycin, moxifloxacin and linezolid. The in vitro drug susceptibility for these drugs were evaluated with a broth microdilution method, and their breakpoint values were followed the recommendation from CLSI [13].

### Statistical analysis

All collected data were entered using Epi Data version 3.1 (EpiData Association, Odense, Denmark). Each entry was cross checked independently to ensure the data quality. The predictor variables of age, sex, residence, previous history for tuberculosis, and comorbidity were tested for association with various NTM diseases using univariate and multivariate logistic analysis. The level of significance of univariate analysis was 0.05, and that for inclusion in the multivariate model was 0.15. Association between NTM diseases and predictor variables was calculated using adjusted odds ratio and 95% confidence interval. In addition, comparison of rate of drug resistance between different NTM species was evaluated by chi-square and Fisher’s exact tests. Differences were considered to be statistically significant at *P* < 0.05. We conducted analyses by using SPSS version 20.0 (SPSS Inc., Chicago, USA).

## Results

### Proportion of different NTM species

**Fig. 1:**
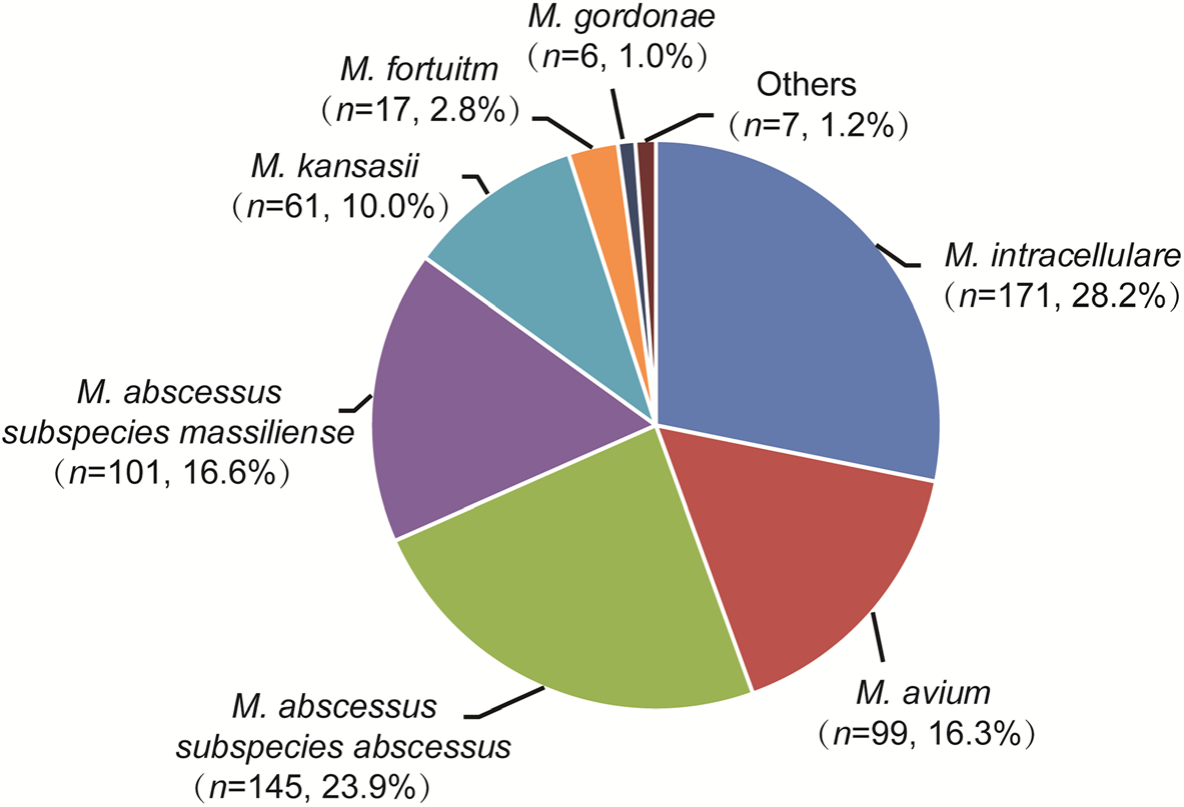
Distribution of nontuberculous mycobacteria species isolated from pulmonary NTM patients in South China

A total of 607 pulmonary NTM patients were enrolled during January 1, 2013-December 31, 2017. Among the 607 NTM cases, the most prevalent species were *M. avium* complex (MAC, 270 isolates, 44.5%), *M. abscessus* complex (MABC, 246 isolates, 40.5%), *M. kansasii* (61 isolates, 10.0%) and *M. fortuitum* (17 isolates, 2.8%). These four groups accounted for 97.9% of all mycobacteria identified. Of 270 *M. avium* complex isolates, there were 171 *M. intracellulare* (63.3%, 171/270) and 99 *M. avium* (36.7%, 99/270) isolates, respectively. In addition, 58.9% (145/246) of *M. abscessus* complex isolates were *M. abscessus subspecies abscessus*, and the remining 41.1% (101/246) belonged to *M. abscessus subspecies massiliense* (Fig. 1).

### Factors associated with SGM and RGM infections

Comparison in demographic and clinical characteristics of NTM patients between SGM and RGM is summarized in Table 1. The male:female ratio was significantly lower among patients infected with RGM than among those with SGM [adjusted odds ratio (aOR): 0.526, 95% confidence interval (95% CI): 0.429–0.862; *P* = 0.005]. In addition, the risk of developing RGM disease significantly decreased with advancing age. Compared with patients with SGM, the adjusted odds ratios were 0.488 (95% CI: 0.287–0.827) for 40–60 years group and 0.395 (95% CI: 0.235–0.666) for > 60 years group, respectively. We also found that the prevalence of infection caused by RGM and SGM differed significantly in resident and migrant population, and pulmonary RGM diseases were more common in migrant population than resident population (aOR: 1.551; 95% CI: 1.092–2.202; *P* = 0.014). Notably, patients with pulmonary RGM diseases were significantly more likely to have bronchiectasis underlying noted than those with SGM diseases (aOR: 1.521; 95% CI: 1.064–2.176; *P* = 0.021). In contrast, there were no other differences regarding TB history or comorbidities noted between SGM and RGM, respectively (*P* > 0.05).

**Table 1:** Comparison in demographic and clinical characteristics of NTM patients between slowly growing mycobacteria and rapidly growing mycobacteria at Guangzhou Chest Hospital, China, January 1, 2013 to December 31, 2017

Characteristics	No. of pulmonary NTM cases (%)		Univariate analysis		Multivariate analysis	
	SGM (*n* = 344)	RGM (*n* = 263)	OR (95% CI)	*P* value	OR (95% CI)	*P* value
Gender						
Female	170 (49.4)	171 (65.0)	0.526 (0.378–0.731)	< 0.001	0.608 (0.429–0.862)	0.005
Male	174 (50.6)	92 (35.0)	1.000	–	1.000	–
Age group (years)						
18–40	34 (9.9)	53 (20.2)	1.000	–	1.000	–
40–60	122 (35.5)	106 (40.3)	0.557 (0.337–0.922)	0.023	0.488 (0.287–0.827)	0.008
> 60	188 (54.7)	104 (39.5)	0.355 (0.217–0.581)	< 0.001	0.395 (0.235–0.666)	< 0.001
TB history						
No	149 (43.8)	96 (36.5)	1.000	–		
Yes	195 (57.4)	167 (63.5)	1.329 (0.953–1.852)	0.093		
Population						
Residence	168 (48.8)	89 (33.8)	1.000	–	1.000	–
Migration	176 (51.2)	174 (66.2)	1.855 (1.331–2.585)	< 0.001	1.551 (1.092–2.202)	0.014
Diabetes						
No	328 (95.3)	252 (95.8)	1.000	–		
Yes	16 (4.7)	11 (4.2)	0.895 (0.408–1.962)	0.895		
Bronchiectasis						
No	158 (45.9)	93 (35.4)	1.000	–	1.000	–
Yes	186 (54.1)	170 (64.6)	1.553 (1.116–2.16)	0.009	1.521 (1.064–2.176)	0.021
COPD						
No	326 (94.8)	256 (97.3)	1.000	–		
Yes	18 (5.2)	7 (2.7)	0.495 (0.204–1.204)	0.121		
Tumor						
No	321 (93.3)	251 (95.4)	1.000	–		
Yes	23 (6.7)	12 (4.6)	0.667 (0.326–1.367)	0.269		

*SGM* slowly growing mycobacteria, *RGM* rapidly growing mycobacteria, *COPD* chronic obstructive pulmonary disease, *OR* odds ratio, *95% CI* 95% confidence interval

### In vitro drug susceptibility profiles of MAC and MABC

We further analysed the in vitro drug susceptibility profiles of *M. avium* complex and *M. abscessus* complex. As shown in Table 2, clarithromycin was the most highly active agent against *M. avium* complex, and the percentages of resistant strains were 4.2% (4/95) for *M. avium* and 3.8% (6/159) for *M. intracellulare*, respectively. Moxifloxacin and linezolid also showed potent activity against *M. avium* complex. There were 5 (5.3%) *M. avium* isolates and 8 (5.0%) *M. intracellulare* isolates resistant to moxifloxacin. For linezolid, the proportions of resistant isolates were 11.6% (11/95) for *M. avium* and 8.2% (13/159) for *M. intracellulare*, respectively. Of the antimicrobial agents tested, amikacin, clarithromycin, linezolid and tobramycin showed highly active against *M. abscessus* complex, and less than 5% of *M. avium* and *M. intracellulare* were resistant to each drug, respectively. In addition, cefoxitin had moderate activity against *M. abscessus* complex, and the percentages of cefoxitin -resistance were observed in 33.8% (46/136) of *M. abscessus subspecies abscessus* and 25.3% (24/95) of *M. abscessus subspecies massiliense* isolates. Statistical analysis revealed that there were no significant differences in the drug resistant rate between *M. abscessus subspecies abscessus* and *M. abscessus subspecies massiliense* (*P* > 0.05) (Table 3).

**Table 2:** Comparison of in vitro drug susceptibility profiles between *M. intracellulare* and *M. avium* isolates

Antimicrobial agents	No. of resistant isolates (%)		*P* value
	*M. avium* (*n* = 95)	*M. intracellulare* (*n* = 159)	
Clarithromycin	4 (4.2)	6 (3.8)	1.000
Moxifloxacin	5 (5.3)	8 (5.0)	1.000
Linezolid	11 (11.6)	13 (8.2)	0.370

The breakpoints to establish susceptibility and resistance for clarithromycin, moxifloxacin and linezolid were followed as recommendation from Clinical and Laboratory Standards Institute (CLSI-M24-A2)

**Table 3:** Comparison of in vitro drug susceptibility profiles between *M. abscessus subspecies abscessus* and *M. abscessus subspecies massiliense* isolates

Antimicrobial agents	No. of resistant isolates (%)		*P* value
	*M. abscessus subspecies abscessus* (*n* = 136)	*M. abscessus* *subspecies massiliense* (*n* = 95)	
Amikacin	3 (2.2)	2 (2.1)	1.000
Clarithromycin	6 (4.4)	3 (3.2)	0.740
Linezolid	6 (4.4)	2 (2.1)	0.476
Tobramycin	6 (4.4)	2 (2.1)	0.476
Cefoxitin	46 (33.8)	24 (25.3)	0.164
Ciprofloxacin	86 (63.2)	68 (71.6)	0.186
Doxycycline	127 (93.4)	94 (98.9)	0.050
Imipenem	134 (98.5)	95 (100.0)	1.000

The breakpoints to establish susceptibility and resistance for drugs were followed as recommendation from Clinical and Laboratory Standards Institute (CLSI-M24-A2)

## Discussion

Pulmonary NTM disease is of increasing public health concern worldwide [6]. This study firstly describes the demographic and clinical characteristics of patients with pulmonary NTM disease in Southern China. Our study has demonstrated that the most common NTM that causes pulmonary disease in Southern China is *M. avium* complex, accounting for 44.5% of pulmonary NTM disease burden in this study, which is consistent with its predominance in other parts of the world, including the United States (85%) [14], Denmark (81%) [15] and South Korea (48%) [16]. The second most frequently identified NTM specie is *M. abscessus* complex. Despite occurring less frequently in Northern America and Europe [14, 15], this species was found to generally cause > 30% of pulmonary NTM infections in India [17], Taiwan [18] and South Korea [16]. The marked geographic variation in mycobacteria species could reflect the diversity of species composition of NTM in environmental niches [3]. Interestingly, all the regions with high isolation frequency of *M. abscessus* are in Asia; we thus speculate that Asian persons may also be more susceptible to *M. abscessus* infection. In line with our hypothesis, Adjemian and colleagues found that Asian persons have an increased risk for infection with *M. abscessus* than other ethnics [19]. Hence, the ethnic factors contributing to susceptibility to different NTM species may also play an important role in the diverse geographic NTM patterns across world regions.

Another interesting finding of this study is that the frequency of NTM species from pulmonary patients differs significantly from the observations from a recent laboratory-based study in Guangzhou [1]. First, *M. avium* complex exceeds *M. abscessus* complex as the predominant causative agent of pulmonary NTM disease, which reflects the inherent difference in NTM prevalence between pulmonary diseases and colonization. The species difference of pulmonary NTM colonization may partly determine the frequency and manifestations of pulmonary NTM disease [3]. However, the variation of pathogenicity among distinct NTM species could greatly contribute to the prevalence of diseases due to NTM species [20]. A population-based study of patients with respiratory NTM isolates from the Netherlands revealed that differences in clinical relevance exist among NTM species [20]. Similar results were noted in a systematic review in Eastern Asian that *M. avium* complex was clinically more relevant than *M. abscessus* complex among patients meeting the ATS diagnostic criteria [21]. Although experimental evidences are limited, there is no doubt that the relative greater pathogenicity of *M. avium* complex compared with *M. abscessus* complex would increase the risk from NTM colonization to active disease in respiratory tract.

Second, *M. gordonae* was the third frequently isolates species in South China on the basis of previous data, accounting for 22.5% of NTM isolates [1]. In contrast, only 1% of pulmonary NTM diseases was caused by *M. gordonae* in the present study. This finding confirms that this species is a rarely isolated weak pathogen, majorly contributing to patient colonization and culture contamination rather than patient disease [3].

Third, several rare geographically restricted NTM species identified by previous study was not associated with NTM diseases [1]. Although for these species the small number of isolates decrease the reliability of conclusions, the disappearance of previous laboratory isolation of these rare species among NTM patients may reflect NTM colonization due to their weak pathogenicity for human individuals. Therefore, the significant change in the prevalence of NTM species between pulmonary diseases and colonization indicates that the pathogenicity differs by species, thereby leading to the difference in clinical relevance of the various NTM species, which should be taken into consideration in formulating the diagnostic criteria for pulmonary NTM diseases.

Pulmonary NTM disease is not uncommon, particularly among elderly females [22]. Our results demonstrate that female is more likely to be associated with the acquisition of NTM diseases caused by RGM than SGM. In agree with our observation, an early study from the United States suggested that *M. avium* complex lung disease was more common among males than females [23]. The gender difference in the NTM diseases may reflect differences in the immune responses during infection between RGM and SGM [24]. Generally, females exhibit greater cell-mediated immune responses to infection and vaccination than males [25], which is also important in host defense against mycobacteria. We therefore hypothesize that this instinct immunological difference may affect in SGM infections more than RGM infections.

Numerous studies have documented that pulmonary NTM infection more frequently affect elderly patients [6, 25]. In this study, we found that the prevalence of SGM increased significantly with advancing age. Furthermore, half of SGM diseases occurred in patients > 60 years of age. On one hand, the immunity in elderly persons is less able to produce an effective immune response after challenges with mycobacteria than the young [26]. This condition would result in greater incidence or reactivation of mycobacteria. On the other hand, the high incidence of co-morbidities presumed to affect the immune response in this population, such as diabetes, kidney failure, and immunosuppressive therapy, may favor the progression of pulmonary NTM infection. More studies are need to evaluate the relative contribution of each factor to the increased risk of pulmonary SGM diseases.

The association between bronchiectasis and NTM disease has been described by several reports [27–29]. We found that the presence of bronchiectasis appears to be more closely associated with RGM than SGM. It has been hypothesized that the impaired secretion clearance due to bronchiectasis enables NTM airway colonization and increases the risk of infection [29]. A recent report by Williams et al. compared the biofilm formation between RGM and SGM, demonstrating that *M. avium* complex is better equipped to grow in low-nutrient conditions than RGM by the development of more culturable biofilm [30]. Although the exact reason remains unknown, the reduced capability in the synthesis of biofilm of RGM allows these species prefer to inhabit in architectural-defected airway rather than normal airway, offering an explanation for the greater occurrence of bronchiectasis among pulmonary RGM cases.

This study is subject to several limitations. First, HIV infection has been regarded as an independent risk factor for NTM infections [31], while the HIV-positive patients were not included in this study because the HIV-positive patients with were transferred to another hospital receiving antiviral treatment. Given that *M. avium* infections are frequently encountered in AIDS patients [31], we may underestimate the prevalence of *M. avium* isolates in Southern China. Second, the clinical outcomes of pulmonary NTM patients were not collected in this study, because patients are not under follow-up for NTM diseases in China. As a consequence, we only analysed in vitro antibiotic susceptibility of NTM isolates rather than its correlation with treatment results. Therefore, further study is urgently needed to investigate the correlation between in vitro drug susceptibility and clinical outcomes among NTM patients. Third, another important explanation for poor response to macrolide-based chemotherapy for *M. abscessus* infections is the inducible macrolide resistance phenotype. Unfortunately, the routine detection of drug susceptibility for RGM only incubates 96-well microtiter plates for 3 days, whereas the detection of inducible resistance requires an extended incubation of plates with reading after 14 days of incubation. As a consequence, the resistance to clarithromycin for *M. abscessus* would be underestimated. Fourth, although cavitary is another major category regarding NTM pulmonary diseases, the radiological characteristics were not collected in this study due to the limited information in the medical records of patients. Nevertheless, this study provides important hints to help clinicians interpret laboratory results and recognize the risk factors associated with various NTM species.

## Conclusion

In conclusion, our data illustrate that the *M. avium* complex is the most predominate causative agent of pulmonary NTM disease in Southern China. Female, migrant population, the presence of bronchiectasis are independent risk factors for pulmonary diseases due to RGM. In addition, the prevalence of SGM increases significantly with advancing age. In view of the growing public health concern, further studies will be carried out to determine the association between in vitro susceptibility and treatment outcome among these NTM patients, which is essential to help clinicians select effective regimens for the treatment of NTM infections.
